# Advances in Computational and Experimental Approaches to Chemical Short‐Range Order Formation in Multi‐Principal Element Alloys

**DOI:** 10.1002/advs.77057

**Published:** 2026-08-06

**Authors:** Mohsen Asle Zaeem, Peter K. Liaw

**Affiliations:** ^1^ Department of Materials Science and Engineering The University of Tennessee Knoxville Tennessee USA

## Abstract

Chemical short‐range order (CSRO) in Multi‐Principal Element Alloys (MPEAs) arises from non‐random atomic arrangements within local coordination shells and plays a critical role in governing mechanical properties, diffusion kinetics, and phase stability. Capturing CSRO requires synergistic computational and experimental strategies. Advanced first‐principles methods evaluate mixing enthalpies and local distortions, while atomistic simulations enable large‐scale configurational sampling. Analytical frameworks, such as the Cluster Variation Method, predict temperature‐dependent ordering trends. Concurrent experimental approaches, including pair distribution function analysis, in situ synchrotron diffraction, atom probe tomography, and resistivity measurements, provide indirect yet complementary insights into local ordering. However, the field lacks a unified framework linking the thermodynamic origin, kinetic evolution, and experimental observability of CSRO, limiting the ability to systematically interpret and compare different approaches. This perspective examines computational and experimental methods in the context of CSRO formation, evolution, and measurement, emphasizes the importance of modeling–experiment cross‐validation pathways for integrating these complementary approaches, and provides a critical assessment of their capabilities, limitations, and roles in advancing the understanding and design of MPEAs.

## Graphical abstract

Chemical short‐range order (CSRO) plays a central role in governing the behavior of multi‐principal element alloys. This perspective critically assesses computational and experimental approaches for quantifying, characterizing, and validating CSRO, emphasizing modeling–experiment cross‐validation pathways that enable more reliable interpretation of local chemical order and accelerate the design of next‐generation alloys.

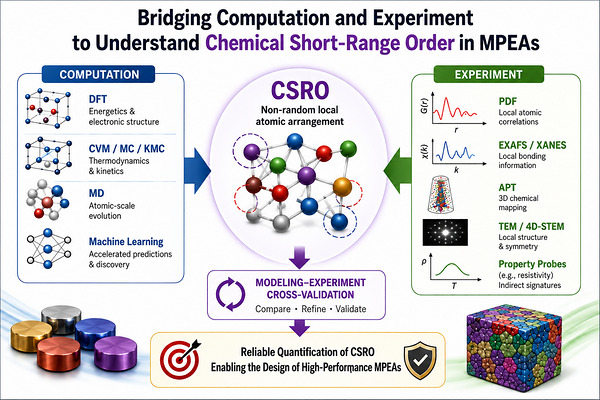

## 1 Introduction

Multi‐Principal Element Alloys (MPEAs), also referred to as High‐entropy Alloys (HEAs) or Medium‐entropy Alloys (MEAs), are defined by their near‐equiatomic mixtures of five or more principal metallic elements, achieving elevated configurational entropy that thermodynamically stabilizes simple solid‐solution phases [face‐centered‐cubic (FCC), body‐centered‐cubic (BCC), and hexagonal‐close‐packed (HCP)] over ordered intermetallic compounds [1, 2, 3, 4]. The pioneering work on the MPEA CoCrFeMnNi in 2004 demonstrated exceptional combinations of high strength, ductility, and thermal stability, igniting broad interest in MPEAs for extreme‐environment applications, such as aerospace, nuclear reactors, and energy‐conversion systems [2]. Early assumptions of ideal atomic randomness have been supplanted by the evidence of chemical short‐range order (CSRO), where statistically significant deviations in atomic‐pair frequencies within the first three neighbor shells modulate fundamental materials behaviors [5, 6, 7, 8].

Importantly, CSRO does not correspond to a unique atomic structure, but rather represents a statistical bias in local chemical environments that can be quantified using a variety of descriptors and inferred through multiple experimental observables. Several descriptors have been developed to quantify CSRO in MPEAs, each emphasizing different aspects of local chemical order. Warren–Cowley parameters are the most widely used descriptors and quantify pair‐wise deviations from random atomic distributions within specific coordination shells, often providing a sufficient description when local ordering is dominated by nearest‐neighbor chemical preferences and pair interactions. Pair‐correlation functions provide a continuous description of chemical correlations in real space, whereas higher‐order cluster correlations capture triplet and multi‐site ordering tendencies that become increasingly important when competing interactions or complex ordering phenomena cannot be adequately described by pair statistics alone. More recently, motif‐based descriptors and machine‐learning representations have emerged to characterize complex local atomic environments and hidden ordering patterns that are difficult to represent using conventional correlation functions. These descriptors are quantified using complementary computational approaches and inferred from experimental observations, which are discussed in detail in Sections 2 and 3. The choice of descriptor also influences the interpretation of experimental observations and cross‐validation strategies. Pair‐wise descriptors are often directly related to average trends observed through scattering‐based techniques and atom probe tomography, whereas higher‐order and motif‐based descriptors may be required to interpret complex local environments revealed by advanced microscopy techniques and local atomic‐environment analyses. Together, these descriptors provide complementary perspectives on CSRO and form an important link between computational predictions and experimental observations. Consequently, robust characterization and validation of CSRO often benefit from combining multiple descriptors and observables, as different atomic configurations may produce similar experimental signatures and no single experimental measurement generally provides a unique determination of the underlying local‐ordering state.

CSRO influences local bonding landscapes by promoting or inhibiting specific atomic pairs, thereby altering stacking‐fault and twin‐boundary energies, modifying solute‐dislocation interactions, and governing vacancy formation and migration energetics [3, 4]. These nanoscale ordering phenomena impact macroscopic properties, including yield strength, work‐hardening rates, creep resistance, and diffusion‐controlled phase transformations. Understanding the thermodynamics and kinetics of CSRO formation is therefore essential for tailoring MPEA performance under service temperatures and mechanical loads.

**FIGURE 1:**
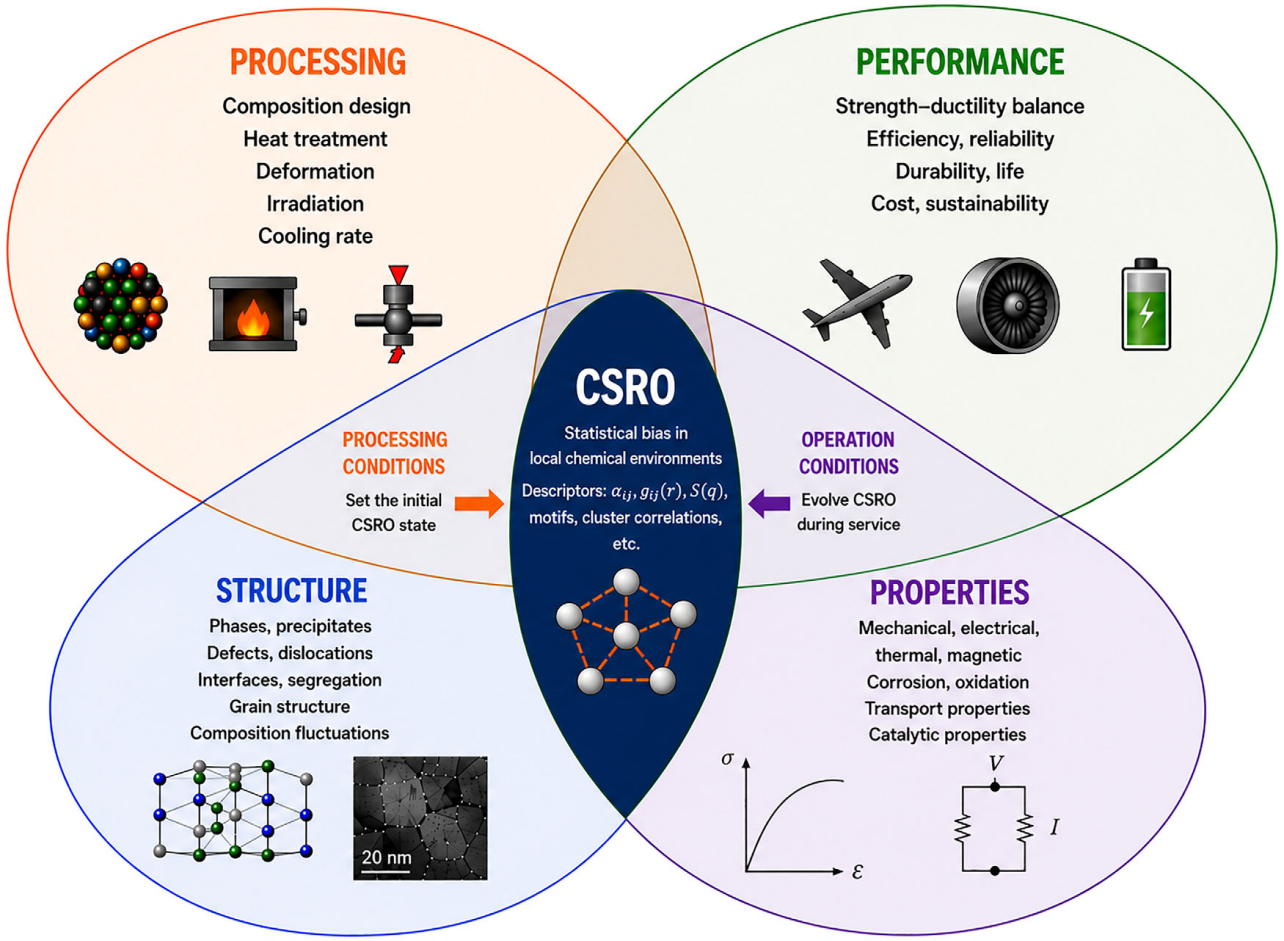
Schematic illustration of the role of CSRO within the PSPP framework for MPEAs.

Despite the recent progress, the field still lacks a unified framework that connects the thermodynamic driving forces for CSRO, its kinetic evolution under processing conditions, and the experimental observables used to infer it. CSRO occupies a central position within the Process–Structure–Property–Performance (PSPP) framework for MPEAs (Figure 1). Processing conditions, including composition design, thermal treatments, deformation, and irradiation, establish local chemical order, while CSRO influences the evolution of microstructural features and, ultimately, material properties and performance. At the same time, external stimuli encountered during operation and service can further modify local chemical environments, making CSRO a dynamic quantity that both responds to and influences the PSPP relationships governing alloy behavior.

Quantifying CSRO in MPEAs remains challenging because no single method can simultaneously resolve its thermodynamic origin, kinetic evolution, and experimentally measurable signatures. Different computational and experimental approaches probe distinct aspects of CSRO, making it difficult to directly compare results or establish consistent interpretations. As a result, progress in the field requires not only the development of advanced methods, but also a clear understanding of their capabilities, limitations, and appropriate domains of applicability. In this work, we present a critical overview of current computational and experimental approaches for studying CSRO in MPEAs, with an emphasis on how these methods complement each other and where important gaps remain.

**FIGURE 2:**
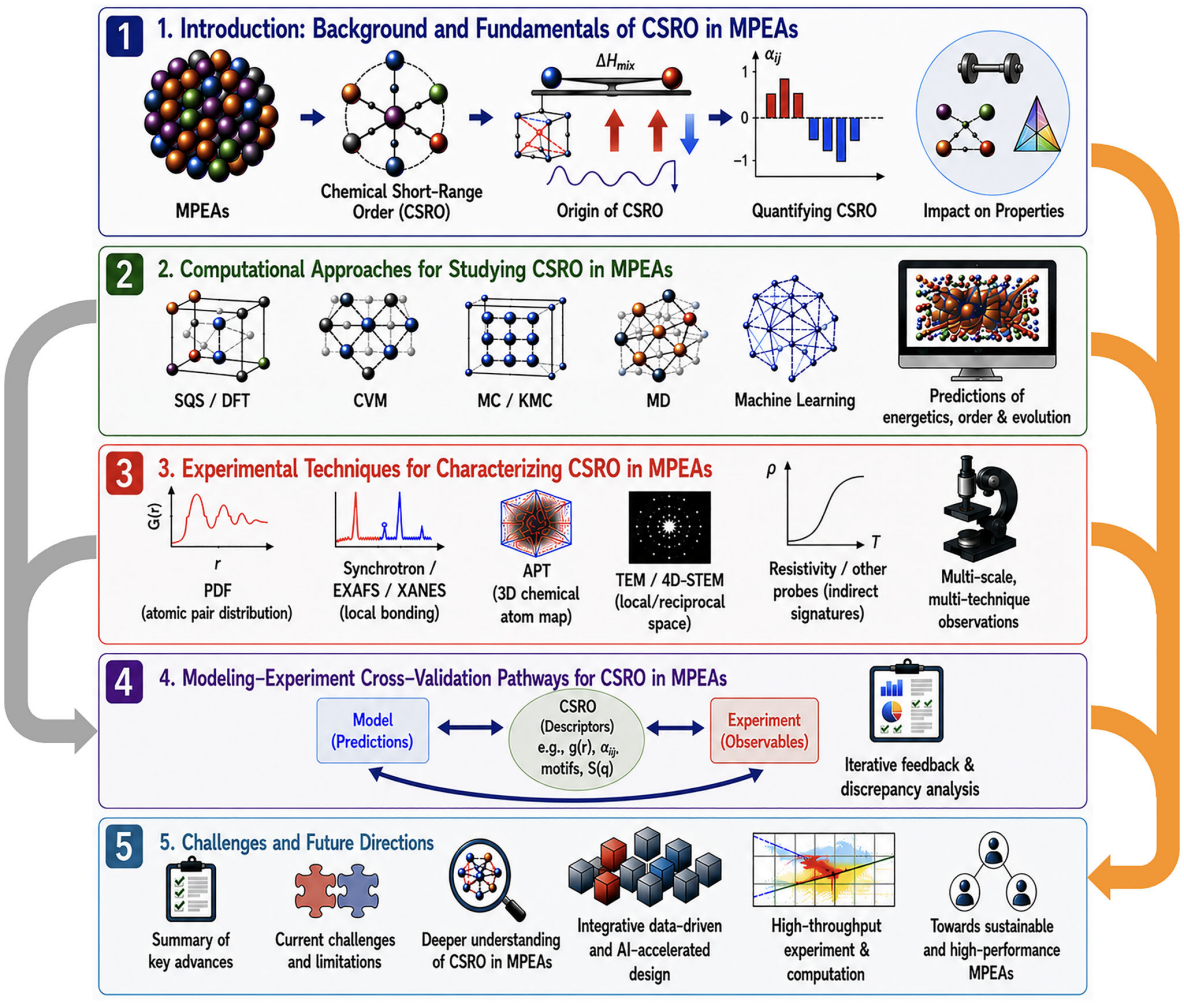
Roadmap of this perspective, illustrating the relationships among fundamental concepts of CSRO, computational approaches, experimental characterization techniques, modeling–experiment cross‐validation pathways, and future research directions.

Figure 2 summarizes the organization and scope of this perspective, beginning with the background and fundamental concepts governing the formation, quantification, and significance of CSRO (Section 1), followed by computational approaches for predicting and quantifying CSRO (Section 2) and experimental techniques for its characterization (Section 3). These complementary computational and experimental methodologies converge through modeling–experiment cross‐validation pathways (Section 4), enabling more reliable interpretation and quantification of local chemical order. The insights gained from these integrated approaches collectively inform current challenges, emerging opportunities, and future research directions for understanding, quantifying, and ultimately utilizing CSRO in the design of next‐generation MPEAs (Section 5).

## 2 Computational Approaches for Studying CSRO in MPEAs

### 2.1 Special Quasi‐Random Structures and Density Functional Theory

Density functional theory (DFT), combined with the Special Quasi‐Random Structure (SQS) method, is widely employed to quantify the thermodynamic driving forces for CSRO by evaluating local chemical energetics in near‐random configurations. The SQS method constructs finite supercells designed to reproduce the pair and multi‐site correlation functions of an ideal random alloy up to a specified coordination shell, thereby providing a practical representation of chemical disorder within a tractable simulation cell. Together, SQS and DFT enable ab initio evaluation of mixing enthalpy, local distortions, and element‐specific interaction energetics in multi‐principal element alloys (MPEAs) [9, 10]. Within this context, SQS + DFT is primarily used to determine whether specific atomic pairs are energetically favored or avoided, thereby providing direct insight into the thermodynamic origin of CSRO.

**FIGURE 3:**
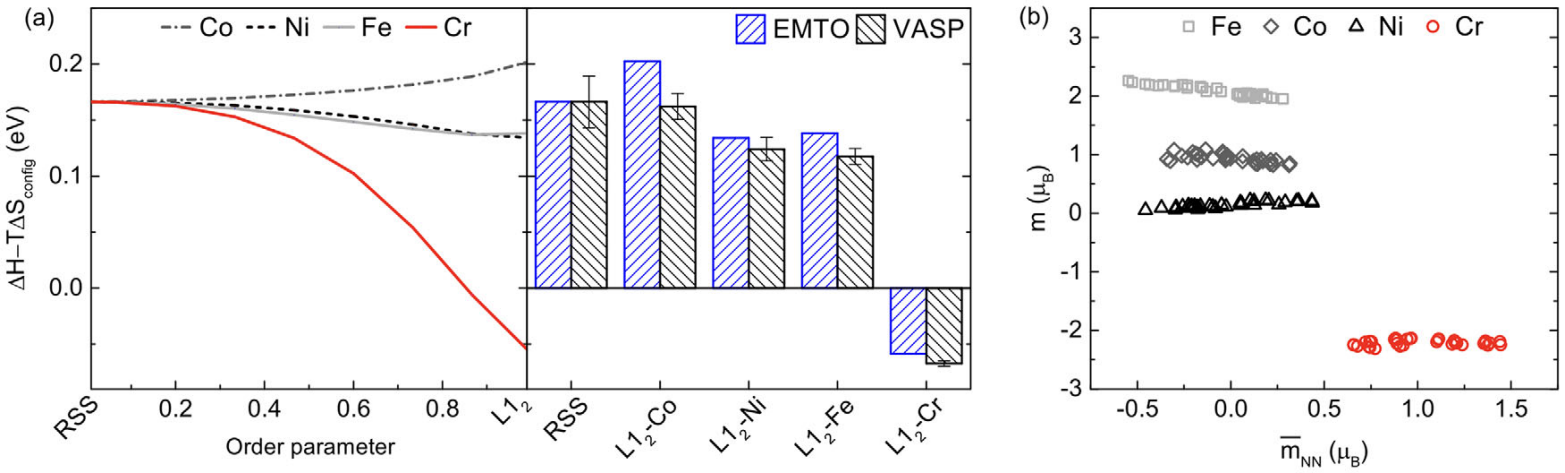
(a) Formation free‐energies of the MPEA NiFeCrCo in random and ordered states at 300 K, and (b) illustration of four possible L1_2_‐type ordering motifs showing Cr‐centric clusters emerging from the SQS‐DFT model. This figure is reprinted from Ref. [13], with permission from AIP Publishing.

Early SQS + DFT studies on ∼100 atom supercells of the prototypical MPEA CoCrFeMnNi reported mixing‐enthalpy differences on the order of a few tens of meV per atom; evidence of Cr‐related ordering was observed in related systems, while finite‐temperature vibrational and magnetic entropy contributions were shown to be essential for quantitative accuracy [11, 12, 13], as shown in Figure 3 for example. Subsequent efforts expanded to ∼200 atom SQS supercells and incorporated additional physics, most notably magnetism and, where relevant, spin–orbit coupling, thereby refining mixing‐enthalpy predictions typically to within a few tens of meV per atom and uncovering element‐specific short‐range ordering in multicomponent systems [14].

More Recently, Machine‐learned (ML) Interatomic Potentials (MLIPs), Trained on Extensive DFT Datasets, Have Enabled SQS‐based Simulations of Several Hundred Atoms (∼200–500) at Substantially Reduced Computational Cost, Achieving Enthalpy Errors on the Order of a Few meV per Atom [15, 16, 17]. In Parallel, High‐throughput SQS Frameworks That Automate the Generation, Relaxation, and Analysis of Supercells Have Been Developed, and When Combined With Quasi‐harmonic and Anharmonic Phonon Free‐energy Calculations, They Improve Finite‐temperature Stability Predictions in MPEAs [18].

Despite these advances, SQS + DFT remains limited in its ability to describe CSRO beyond the predefined local correlations imposed in the SQS construction. The approach relies on finite supercells and does not naturally capture how CSRO evolves with temperature or processing conditions. While temperature effects can be incorporated through additional approximations, such as vibrational or magnetic entropy contributions, they are not treated explicitly within standard SQS + DFT frameworks. In addition, the method cannot directly capture the kinetics of CSRO formation. Therefore, while SQS + DFT is effective for identifying thermodynamic tendencies for ordering, it must be complemented by statistical and kinetic approaches to fully describe CSRO behavior. These limitations motivate the use of methods capable of configurational sampling and time‐dependent evolution, as discussed in the following sections.

### 2.2 Cluster‐Variation Method

The Cluster‐Variation Method (CVM) provides a deterministic statistical‐mechanical framework for quantifying CSRO by expressing the configurational‐free energy in terms of cluster‐occupancy probabilities (pairs, triplets, tetrahedra, etc.) and finding equilibrium by minimizing this free energy under normalization and compatibility constraints [19, 20]. By systematically increasing the cluster size, CVM captures correlations beyond simple mean‐field approximations and can approximate Monte Carlo accuracy at far lower computational cost. In this context, CVM is primarily used to predict equilibrium CSRO as a function of temperature and to evaluate ordering tendencies in multicomponent systems.

**FIGURE 4:**
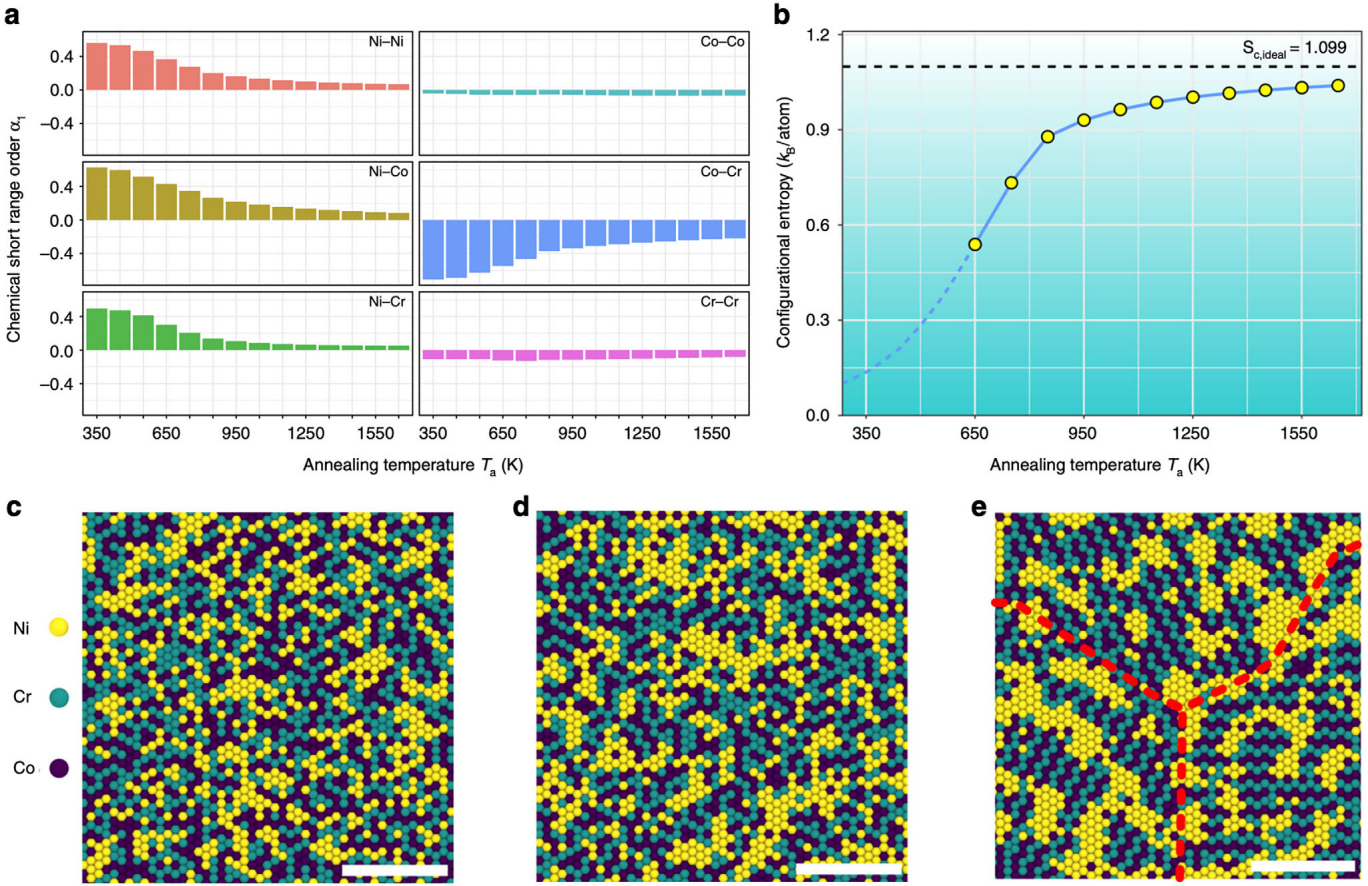
Finite‐temperature evolution of local chemical order in the MPEA NiCoCr. (a) First‐neighbor short‐range‐order parameters (α_1_) for key atomic pairs versus annealing temperature. (b) Configurational entropy from cluster‐variation‐method (CVM) calculations showing reduced randomness on cooling. (c–e) Atomic configurations at 1,350, 950, and 650 K, illustrating the emergence of Co─Cr‐enriched domains and Ni segregation as ordering develops. This figure is reprinted from Ref. [22], licensed under CC BY 4.0.

Limited studies showed the applications of CVM to MPEAs in capturing finite‐temperature ordering phenomena. In the MPEA Cu─Fe─Ni, Luo et al. [21] employed a DFT‐parameterized CVM framework to quantify CSRO and assess its effect on mechanical response. Their results revealed that CSRO synergistically enhances both strength and toughness through the reduced configurational entropy and stabilization of local lattice distortions. Similarly, in the FCC MPEA NiCoCr, Li et al. [22] used a pair‐CVM approximation, combined with molecular‐dynamics simulations and experimental observations, to show the progressive development of CSRO below approximately 700 K, Figure 4. The emergence of CSRO roughens dislocation pathways and increases friction stresses through nanoscale‐dislocation detrapping, thereby explaining the anomalous strengthening observed at intermediate temperatures. In contrast, severe plastic deformation was found to disrupt CSRO, restoring random atomic configurations and lowering lattice resistance to slip.

Building on these early applications [21, 22, 23], high‐throughput CVM pipelines now automate the extraction of cluster‐interaction energies from DFT or Calculation of Phase Diagrams (CALPHAD) databases and solve extensive non‐linear systems for multicomponent alloys, producing CSRO‐temperature maps within tractable runtimes. A recently proposed cluster‐based thermodynamic framework embeds intrinsic CSRO directly into CALPHAD‐type databases [24]. Although it has not yet been applied to MPEAs, adopting such approaches would allow the incorporation of CSRO physics into practical thermodynamic models, enabling quantitative phase‐stability predictions and guiding alloy‐design strategies where CSRO critically impacts mechanical properties.

In earlier work, a hybrid CVM‐phase‐field model was applied to simpler alloys, where the elastic strain energy was incorporated into ordering transitions [25], but there has been little subsequent progress in extending this approach to multicomponent systems. In parallel, phase‐field simulations of MPEAs have successfully reproduced coherent BCC/B2 precipitate morphologies and quantified how the lattice misfit and elastic anisotropy govern their stability and coarsening, though without explicit CSRO thermodynamics [26]. This feature suggests a pathway for the future integration into MPEA frameworks.

Despite these advantages, CVM has important limitations. Its accuracy depends on the choice and truncation of cluster interactions, which may affect predictions in multicomponent systems. In addition, CVM is an equilibrium approach and does not describe the kinetics of CSRO evolution. Therefore, while CVM is effective for predicting equilibrium CSRO trends, it must be complemented by atomistic and kinetic approaches to more fully describe CSRO behavior.

### 2.3 Monte Carlo and Molecular Dynamics Simulations

Monte Carlo (MC) and Molecular Dynamics (MD) simulations provide complementary statistical and atomistic approaches for quantifying CSRO and its effects on thermodynamics, transport, and mechanical behavior in multi‐principal element alloys (MPEAs). MC techniques stochastically sample atomic configurations through site exchanges with Boltzmann‐weighted acceptance probabilities, enabling equilibrium mapping of CSRO trends across temperature and composition. MD simulations, in turn, propagate atomic trajectories under interatomic forces derived from DFT or ML potentials, capturing the dynamic consequences of CSRO on defect energetics, vibrational properties, and deformation pathways. MC is primarily used to determine equilibrium CSRO configurations and temperature‐dependent ordering tendencies, while MD is used to evaluate how established CSRO states influence atomic‐scale mechanisms, such as defect motion and deformation.

In addition to equilibrium MC and atomistic MD approaches, kinetic MC (KMC) provides a critical framework for modeling the time‐dependent evolution of CSRO under diffusion‐controlled processes [27]. KMC explicitly simulates atomistic diffusion events over extended timescales by incorporating transition rates derived from first‐principles calculations or machine‐learned potentials, enabling direct prediction of ordering kinetics, activation energies, and temporal evolution of short‐range order. Recent studies on CrCoNi and NiCoCr alloys have demonstrated that KMC simulations capture the evolution of Warren–Cowley parameters during annealing and yield activation energies consistent with experimental resistivity measurements, establishing a direct link between atomistic diffusion processes and experimentally observable CSRO evolution [27]. Unlike MC, which samples configurational space without explicit time, KMC provides access to realistic kinetic pathways, making it essential for connecting processing conditions to CSRO development in MPEAs.

In equilibrium studies, Fernández‐Caballero et al. applied the cluster‐expansion‐based MC to the MPEA MoNbTaVW refractory system, demonstrating distinct CSRO patterns and their temperature evolution [28]. Andreoli et al. combined first‐principles energetics with MC sampling and showed that CSRO strongly modifies thermophysical properties, such as the thermal expansion and heat capacity in MEAs and HEAs, highlighting the importance of incorporating CSRO into predictive models [29]. Sun et al. studied the MPEA Al_0_._3_CoCrFeNi with MC simulations and found that the emergence of SRO altered dislocation pathways, thereby increasing yield strength while retaining ductility, offering direct atomistic evidence for the role of CSRO in governing MPEA mechanical performance [30]. More recently, ML‐accelerated MC frameworks have scaled to tens of thousands of atoms, producing quantitative CSRO–temperature maps and enabling diffusion barrier predictions [31, 32].

**FIGURE 5:**
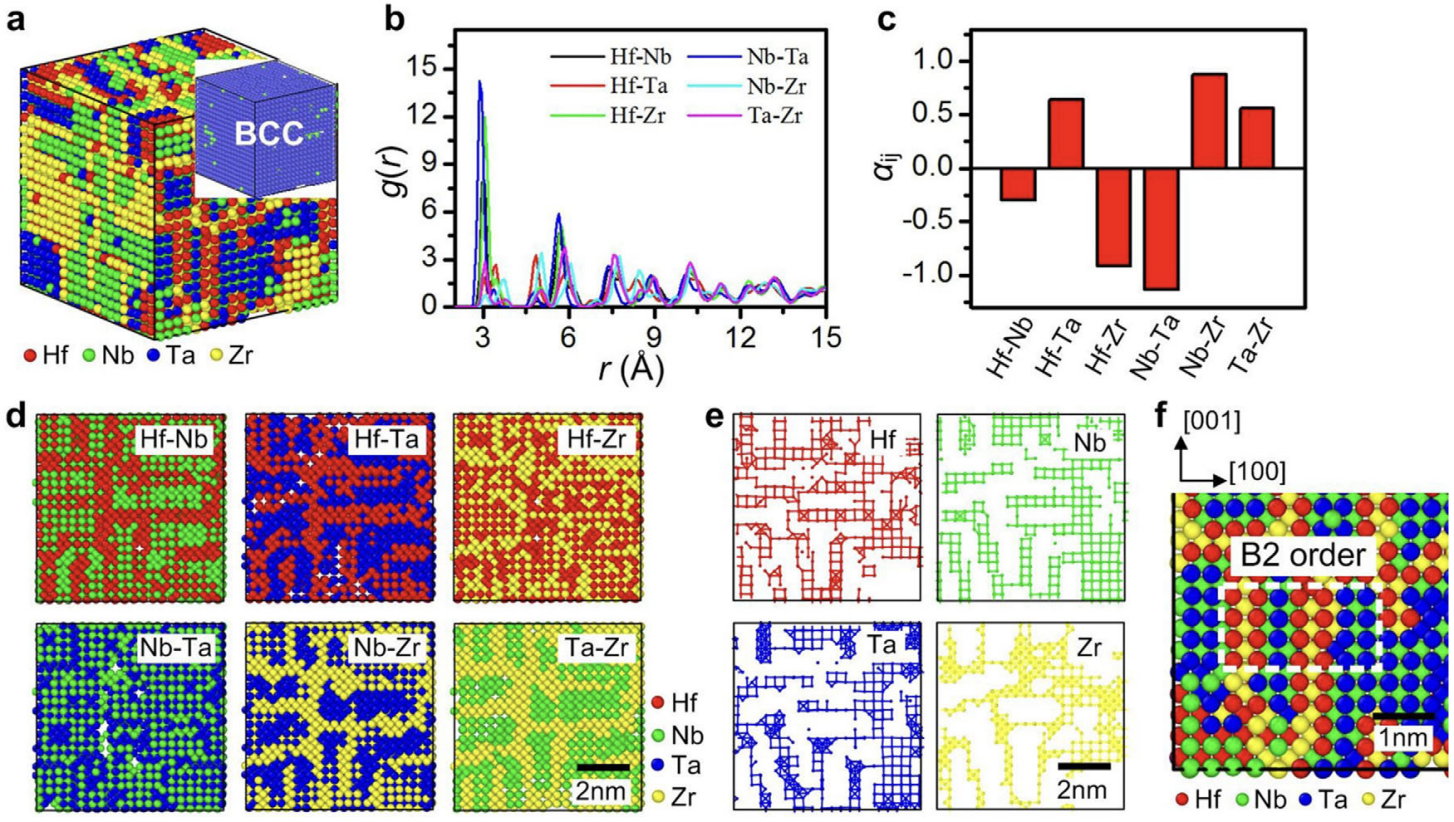
Hybrid MD–MC simulations to study CSRO in the MPEA HfNbTaZr. (a) the atom distribution and atom types. (b) the PPCFs (partial pair correlation functions) for the atoms. The curves have an obvious difference in several nearest‐neighbor shells. (c) The corresponding Warren‐Cowley parameters showing the enrichment of Nb‐Ta and Hf‐Zr pairs in the BCC solid solution, (d) atom distribution of two elements, (e) atom distribution of one element, and (f) atom structure with B2 order. This figure is reprinted from Ref. [34], with permission from Elsevier.

Beyond equilibrium, MD simulations have revealed the dynamic impact of CSRO on defect energetics and mechanical behavior. Ding et al. showed that introducing CSRO reduces the stacking‐fault energy in the MPEA NiCoCr by up to 30%, stabilizing twinning, whereas random chemical states favor dislocation glides [33]. Hybrid MC–MD studies extended this framework: Li, Sheng, and Ma generated CSRO‐configured states via MC equilibration and then conducted MD shear simulations, demonstrating that CSRO roughens the dislocation‐energy landscape, intermittently pinning dislocation segments and enhancing strength without loss of ductility [22]. Hybrid MD–MC has also been applied to refractory MPEAs, where Huang et al. showed in the MPEA HfNbTaZr that CSRO persists across wide temperature ranges and stabilizes specific atomic environments, Figure 5 [34]. More recently, Ghosh et al. used ML interatomic potentials in combined MC/MD sampling of the NiCrCo, achieving supercells of tens of thousands of atoms, and quantitatively mapping CSRO–temperature trends linked to phase stability [31]. Collectively, these studies demonstrate that atomistic approaches can be used not only to assess the effects of CSRO on deformation behavior, but also to investigate the interactions among local chemical order, defect activity, and microstructural evolution during deformation.

Despite these advances, important limitations remain. Conventional equilibrium MC simulations describe equilibrium configurations and temperature‐dependent ordering tendencies but do not provide an explicit physical time scale for CSRO evolution. In contrast, KMC can capture diffusion‐controlled ordering kinetics over extended timescales when reliable event catalogs and transition rates are available. MD simulations remain limited to relatively short timescales (typically picoseconds to nanoseconds), restricting their ability to model slow diffusion‐driven ordering processes directly. In addition, the accuracy of MC, KMC, and MD simulations depends on the quality of the underlying energetic and kinetic models, including cluster expansions, machine‐learning potentials, and transition‐rate parameterizations. Therefore, while these atomistic approaches provide critical insight into equilibrium CSRO, ordering kinetics, and their impact on atomic‐scale mechanisms, additional multiscale approaches are often required to bridge experimentally relevant length and time scales.

Although most existing studies focus on the deformation response of initially ordered configurations, the same atomistic frameworks provide a foundation for investigating deformation‐induced evolution of local chemical environments and their coupling with defect activity. Extending such approaches toward quantitative prediction of CSRO evolution over experimentally relevant length and time scales remains an important challenge.

Together, MC and MD methods demonstrate how CSRO influences defect energetics, thermophysical behavior, and deformation pathways in MPEAs. Collectively, studies on refractory alloys, such as MoNbTaVW [28] and HfNbTaZr [34], as well as Al‐containing systems, such as Al_0_._3_CoCrFeNi [30], show that the nature and consequences of CSRO vary significantly across different alloy families. Monte Carlo simulations reveal composition‐ and temperature‐dependent ordering tendencies, while hybrid MD–MC approaches provide insight into the persistence of CSRO and the stabilization of local atomic environments [34]. In Al_0_._3_CoCrFeNi, CSRO has been shown to alter dislocation pathways and strengthening mechanisms [30]. These observations highlight the strong dependence of CSRO on alloy chemistry and crystal structure. The integration of cluster‐expansion MC with ML interatomic potentials and hybrid MD–MC workflows will further enable predictive simulations at realistic length and time scales, establishing these methods as central tools for unraveling CSRO‐driven mechanisms in complex alloys.

### 2.4 Machine‐Learning (ML) Approaches

The ML approaches have emerged as indispensable surrogates for computationally expensive CSRO models in MPEAs. Physics‐Informed Neural Networks (PINNs) have been proposed to embed thermodynamic free‐energy constraints within the loss function, reducing the volume of required training data while preserving physical consistency [35]. Graph Neural Networks (GNNs), which represent atoms as graph nodes and chemical bonds or neighbor interactions as edges, have been employed to predict the phase stability and CSRO descriptors in MPEAs. Recent work has shown that equivariant GNNs can accurately characterize chemical motifs and local environments in MPEAs, enabling the quantitative assessment of SRO and its impact on alloy stability [36]. Similarly, ML‐accelerated cluster‐expansion frameworks that incorporate GNN models have been used to efficiently generate high‐throughput CSRO–temperature maps across thousands of alloy chemistries, bridging atomistic simulations with thermodynamic modeling [37]. These approaches demonstrate that GNNs can achieve near‐ab initio accuracy while drastically reducing computational cost, making them powerful surrogates for exploring chemical‐ordering phenomena in complex alloys. In addition, these approaches are primarily used to accelerate the prediction of CSRO energetics, local configurations, and temperature‐dependent trends that would otherwise require extensive DFT or MC simulations.

Deep‐learning techniques have also enabled atomistically resolved simulations of local ordering phenomena. For example, Zhang et al. [38] introduced the Deep Potential Molecular Dynamics (DeepMD) framework, which constructs interatomic potentials trained on high‐fidelity DFT data. DeepMD simulations have been used to investigate the CSRO formation and evolution in disordered multicomponent alloys with near‐ab initio accuracy, bridging the gap between the large‐scale configurational sampling and physical realism. Building on this process, Freitas's group [31, 32, 39] demonstrated that properly trained ML interatomic potentials can reliably capture SRO in ternary MPEAs, showing how the choice of training data and descriptors directly controls fidelity to ordering energetics. The combination of ML interatomic potentials with large‐scale atomistic simulations also provides opportunities to investigate local‐order evolution during deformation and service [31, 32, 39]. By extending accessible length and time scales beyond conventional atomistic simulations, these approaches may enable more realistic studies of the coupling between local chemical order, defect activity, and deformation processes.

Despite these advances, current ML frameworks still face important challenges. The transferability of ML models beyond the chemical, configurational, and thermodynamic domains represented in the training data requires careful validation, particularly when extending predictions to new alloy systems, defect configurations, or processing conditions. In addition, uncertainty quantification (UQ) remains relatively uncommon, limiting confidence in predictions of energies, forces, CSRO descriptors, phase stability, and derived materials properties. Furthermore, many ML surrogate models are designed to accelerate equilibrium predictions of CSRO energetics and ordering trends and therefore do not explicitly capture the kinetics of CSRO evolution. However, ML interatomic potentials coupled with molecular dynamics or kinetic Monte Carlo frameworks can, in principle, describe time‐dependent ordering processes when physically meaningful dynamics or transition rates are incorporated.

Table 1 summarizes the computational‐modeling tools available for the study and quantification of CSRO, together with their principal strengths and limitations. These approaches should be viewed as complementary rather than competing methodologies. First‐principles calculations provide the thermodynamic foundations of CSRO by quantifying chemical interactions and energetic driving forces, while statistical thermodynamic approaches such as CVM predict equilibrium ordering tendencies and temperature‐dependent behavior. Monte Carlo and kinetic Monte Carlo simulations extend these capabilities by capturing configurational evolution and ordering kinetics, whereas molecular dynamics enables the investigation of atomistic mechanisms and the influence of CSRO on material properties. More recently, machine‐learning approaches have emerged as powerful tools for extending simulations to larger length and time scales while maintaining near first‐principles accuracy. Consequently, no single computational method is sufficient to fully describe the formation, evolution, and consequences of CSRO, and meaningful progress increasingly relies on integrated multiscale frameworks that combine the strengths of multiple approaches.

**TABLE 1:** Computational methods for CSRO quantification.

Method	Scale	Key Advancements	Limitations	References
SQS + DFT	80 – 500 atoms (DFT); up to 200 – 500 atoms (MLIPs)	Quantifies mixing enthalpy and local distortions; detects element‐specific CSRO; MLIPs extend cell sizes for CSRO analysis	High computational cost; finite‐size limits; temperature effects approximated	[9, 10, 11, 12, 13, 14, 15, 16, 17, 18]
CVM	Lattice clusters	Predicts finite‐T SRO trends; high‐throughput CVM pipelines generate CSRO–T maps; CALPHAD integration emerging	Cluster truncation; neglects kinetics; limited use in multicomponent phase‐field	[21, 22, 23, 24, 25, 26]
Monte Carlo (MC)	4000–125 000 atoms	Maps equilibrium SRO trends; KMC captures ordering kinetics and activation energies; cluster‐expansion + ML acceleration enables large‐scale SRO–T predictions	Slow near Tc; conventional MC lacks an explicit physical time scale; accuracy depends on underlying energetic and kinetic models	[27, 28, 29, 30, 31, 32, 33]
Molecular Dynamics (MD)	∼ 200–1 500 000 atoms	Shows how SRO alters defect energetics and deformation (e.g., SFE, dislocation pathways); hybrid MC–MD improves realism	Short timescales (ps – ns); accuracy tied to interatomic potentials	[22, 31, 33, 34]
Machine‐ Learning (ML) Approaches	Composition‐wide; up to tens of thousands of atoms	PINNs embed CSRO‐related thermodynamic constraints; GNNs predict CSRO motifs/local environments; DeepMD/MLIPs capture CSRO formation and evolution with near‐DFT accuracy	Transferability beyond the training domain requires validation; interpretability issues; UQ rarely reported; equilibrium‐focused models may not capture CSRO kinetics	[31, 32, 35, 36, 37, 38, 39]

Future progress requires standardized FAIR (Findability, Accessibility, Interoperability, and Reusability) datasets with experimentally validated CSRO metrics, physics‐informed architectures that enforce pair/triplet correlation constraints, and active‐learning loops coupling ML with DFT, MC, and in situ characterization. These directions will transform ML models from predictive surrogates into robust design tools for CSRO‐controlled alloy development.

## 3 Experimental Techniques for Characterizing CSRO in MPEAs

### 3.1 Total Scattering and PDF

Total‐scattering techniques that combine Bragg diffraction and diffuse scattering provide the pair distribution function (PDF), a real‐space measure of atomic‐pair correlations. This approach is primarily used to quantify short‐range atomic correlations and detect deviations from ideal random solid‐solution behavior, making it a key experimental probe for CSRO.

**FIGURE 6:**
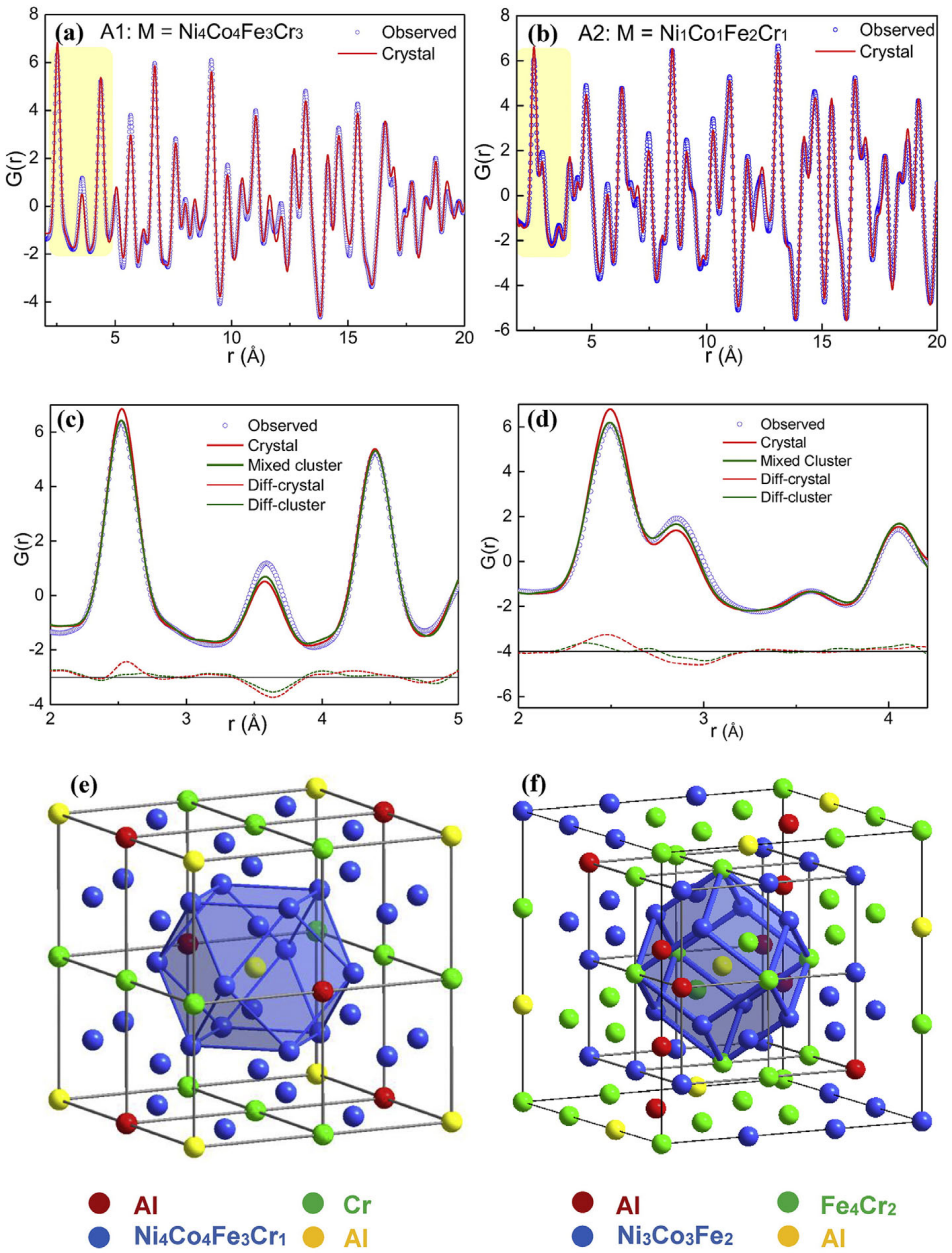
The local structures of Al_2_M_14_ MPEAs. (a) and (b) show the PDFs of A1 (M  =  Ni_1_Co_1_Fe_2_Cr_1_) and A2 (M  =  Ni_1_Co_1_Fe_2_Cr_1_) alloys measured by the neutron scattering, respectively, in which the measured PDFs match well with the calculated PDFs with the average crystal structures at long distances (a, b) that could not fit the measured PDFs at short inter‐atomic distances (the first two peaks) (c, d). (e) and (f) are the cluster‐plus‐glue‐atom models of Al_2_M_14_ in FCC and BCC structures, respectively. This figure is reprinted from Ref. [43], with permission from Elsevier.

Neutron total‐scattering measurements on the MPEA CrMnFeCoNi alloy have shown well‐resolved PDF peaks extending to approximately 5 Å, corresponding to the first several coordination shells. These data confirm a largely random FCC arrangement while enabling the quantitative evaluation of local lattice strains and subtle deviations from perfect periodicity [40]. Similar approaches have been applied to other high‐entropy alloys: PDF analyses of Zr─Nb─Hf systems reveal deviations in the first two coordination shells associated with the atomic‐size mismatch [41], and total‐scattering combined with reverse‐Monte‐Carlo (RMC) modeling in multicomponent HEAs and their deuterides identifies local atomic correlations indicative of emerging short‐range order [42]. Ma et al. extended this use of total‐scattering techniques by showing that CSRO can directly influence local structural states. Using neutron total‐scattering and pair‐distribution‐function analysis, they demonstrated that specific CSRO motifs in Al─Ni─Co─Fe─Cr alloys modify short‐range atomic correlations sufficiently to induce transitions between FCC‐like and BCC‐like local structures. Their cluster‐based structural modeling reproduced the experimental PDFs more accurately than average crystal structures, indicating that CSRO can shift the relative stability of competing phases and act as a precursor to structural transformation pathways in MPEAs [43], as shown in Figure 6. It is found from the featureless error curves in Figure 6c,d that the measured PDFs of MPEAs at a short range within *r* < 5.0 Å could be matched better with the cluster models than with the average crystal structures, indicating the CSRO in MPEAs.

However, PDF analysis does not generally provide a unique determination of CSRO, as different atomic configurations can produce similar pair‐correlation signatures. Therefore, PDF‐based characterization should be interpreted in conjunction with complementary techniques or modeling to more reliably identify CSRO motifs. The examples discussed above, spanning CrMnFeCoNi [40], Zr─Nb─Hf [41], and Al─Ni─Co─Fe─Cr alloy systems [43], demonstrate that PDF analysis can reveal CSRO‐related structural signatures across chemically diverse MPEAs with distinct local bonding environments and crystal structures.

### 3.2 High‐Energy Synchrotron X‐Ray Diffraction and Extended X‐Ray Absorption Fine Structure (EXAFS)

In situ high‐energy synchrotron X‐ray diffraction during annealing and non‐equilibrium solidification has been used to track the evolution of ordering and phase stability in high‐entropy alloys. These approaches are primarily used to monitor phase selection and ordering during processing, providing indirect constraints on CSRO through time‐ and temperature‐dependent structural changes. Reynolds et al. combined CALPHAD thermodynamic predictions with in situ high‐energy synchrotron XRD on the MPEA Al_0_._3_CoCrFeNi, following the emergence and partial dissolution of FCC, B2, σ, and L1_2_ phases during multi‐hour anneals and demonstrating that multiphase HEAs can remain far from equilibrium on experimental time scales [44]. Andreoli et al. used containerless electromagnetic levitation with the high‐energy synchrotron XRD to obtain time‐ and temperature‐resolved phase formation in the CoCrFeNi and related MEAs/HEAs, revealing the metastable primary BCC solidification, delayed the nucleation of the stable FCC phase, and a strong dependence of transformation pathways on melt undercooling [45]. In a follow‐up study on the Al_x_CoCrFeNi (x = 0.3 and 1), they showed that Al additions modify non‐equilibrium solidification and subsequent ordering: in situ high‐energy synchrotron XRD captures the solidification path, while ex situ XRD reveals the precipitation of ordered L1_2_ phases at modest undercooling, underscoring sluggish kinetics and path‐dependent phase selection in Al‐containing HEAs [46]. These in situ diffraction data provide stringent benchmarks for models of CSRO formation and evolution because any proposed CSRO pathway must be compatible with the experimentally observed time–temperature windows for phase formation and ordering.

**FIGURE 7:**
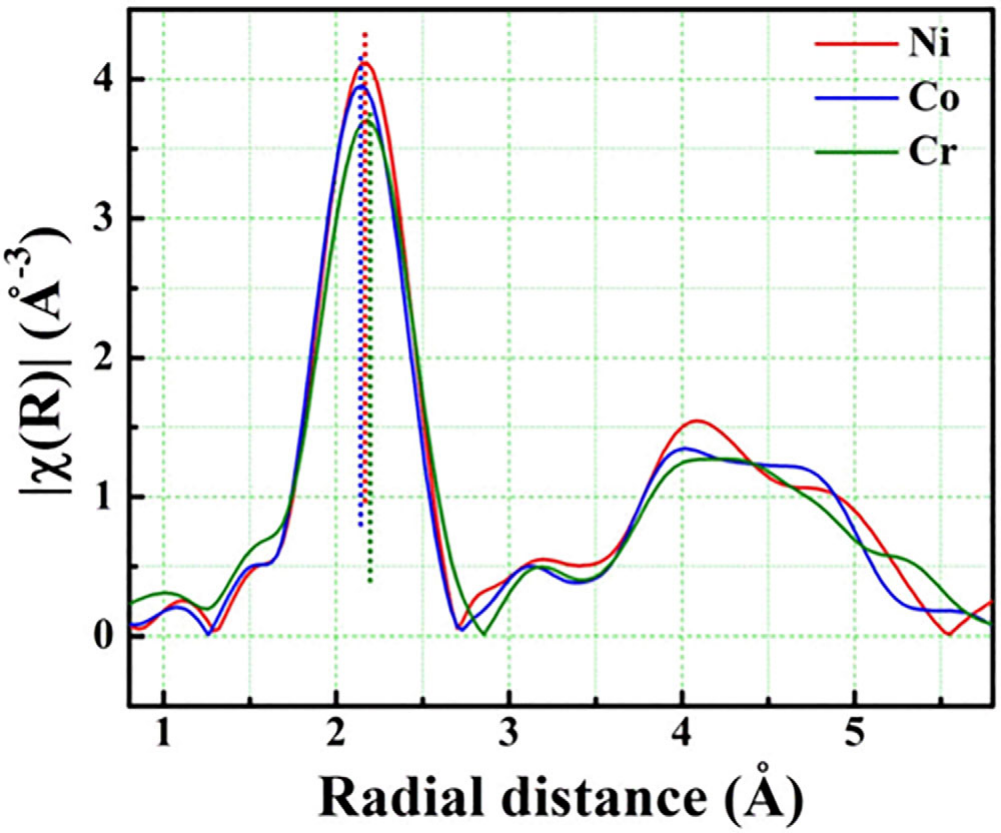
Magnitude of EXAFS functions measured in the 𝐾 edge of Ni, Co, and Cr in the MPEA NiCoCr and the slight difference in the position of the first peak suggests that the bond length of Ni, Co, and Cr with its surrounding atoms could be different. This figure is reprinted from Ref. [47], with permission from the American Physical Society.

In addition, the extended x‐ray absorption fine structure (EXAFS) could be used to indicate the presence of CSRO, as shown in Figure 7 [47]. Figure 7 presents the magnitude of the EXAFS function in real space of the NiCoCr. The first shell peaks around Ni, Co, and Cr have a somewhat mismatch, which indicates that the local bond length between different atomic species could slightly vary. To fit the EXAFS, a cluster in a fully disordered occupation in an FCC lattice was built based on the Warren‐Cowley parameter calculation [48]. It was found that the NiCoCr was not a fully disordered system. There is short‐range order, which suggests that Cr atoms have a tendency to separate each other, and more Ni(Co)—Cr pairs are present [47].

However, both high‐energy synchrotron XRD and EXAFS provide indirect evidence of CSRO rather than a unique or direct measurement of CSRO. High‐energy synchrotron XRD is primarily sensitive to phase evolution and average structural changes, while its sensitivity to short‐range chemical correlations can be limited when diffuse scattering is weak. Similarly, EXAFS probes local atomic environments, but its interpretation depends on structural modeling and fitting assumptions. Therefore, these techniques are most effective when combined with complementary experimental methods or modeling to more reliably assess CSRO.

### 3.3 Atom‐Probe Tomography (APT)

The APT reconstructs 3D atomic‐scale composition maps with near‐atomic spatial resolution, enabling the direct analysis of local chemical environments and, under appropriate conditions, quantitative CSRO metrics. This technique is used to probe local compositional fluctuations and short‐range atomic correlations, providing one of the most direct experimental probes of CSRO in MPEAs. He et al. developed a framework to quantify CSRO from APT datasets in compositionally complex alloys, mapping how detector efficiency and spatial resolution control the minimum measurable CSRO and defining a go/no‐go threshold for reliable Warren–Cowley–type parameters in the MPEA NiCoCr [49]. In the NiCoCr, Inoue et al. used APT to directly observe nanoscale CSRO: along the [001] direction, Cr‐rich and (Ni + Co)‐rich {001} atomic layers alternate over approximately ten atomic planes (∼ 2 nm), whereas no such modulation appears along [111]. They also showed that this layered CSRO has only a limited influence on bulk mechanical properties [50]. ML–enhanced reconstruction further extends these capabilities.

Li et al. combined APT with a neural‐network‐based reconstruction scheme to overcome spatial‐resolution limits and achieved quantitative 3D imaging of multiple CSRO motifs in Fe─Al alloys, showing non‐statistical B2‐type CSRO rather than the generally expected D0_3_‐type order and correlating the CSRO state with nano‐hardness and electrical resistivity [51]. Beyond metallic species, interstitial solutes can also participate in ordered complexes: in a TiZrHfNb high‐entropy alloy, Lei et al. used APT together with electron microscopy and diffraction to identify ordered oxygen complexes, that is, nanoscale (O, Zr, Ti)‐rich regions whose formation is promoted by substitutional CSRO and which simultaneously enhance strength and ductility, illustrating how solute–CSRO interactions can be exploited to tune mechanical response in compositionally complex alloys [52].

**FIGURE 8:**
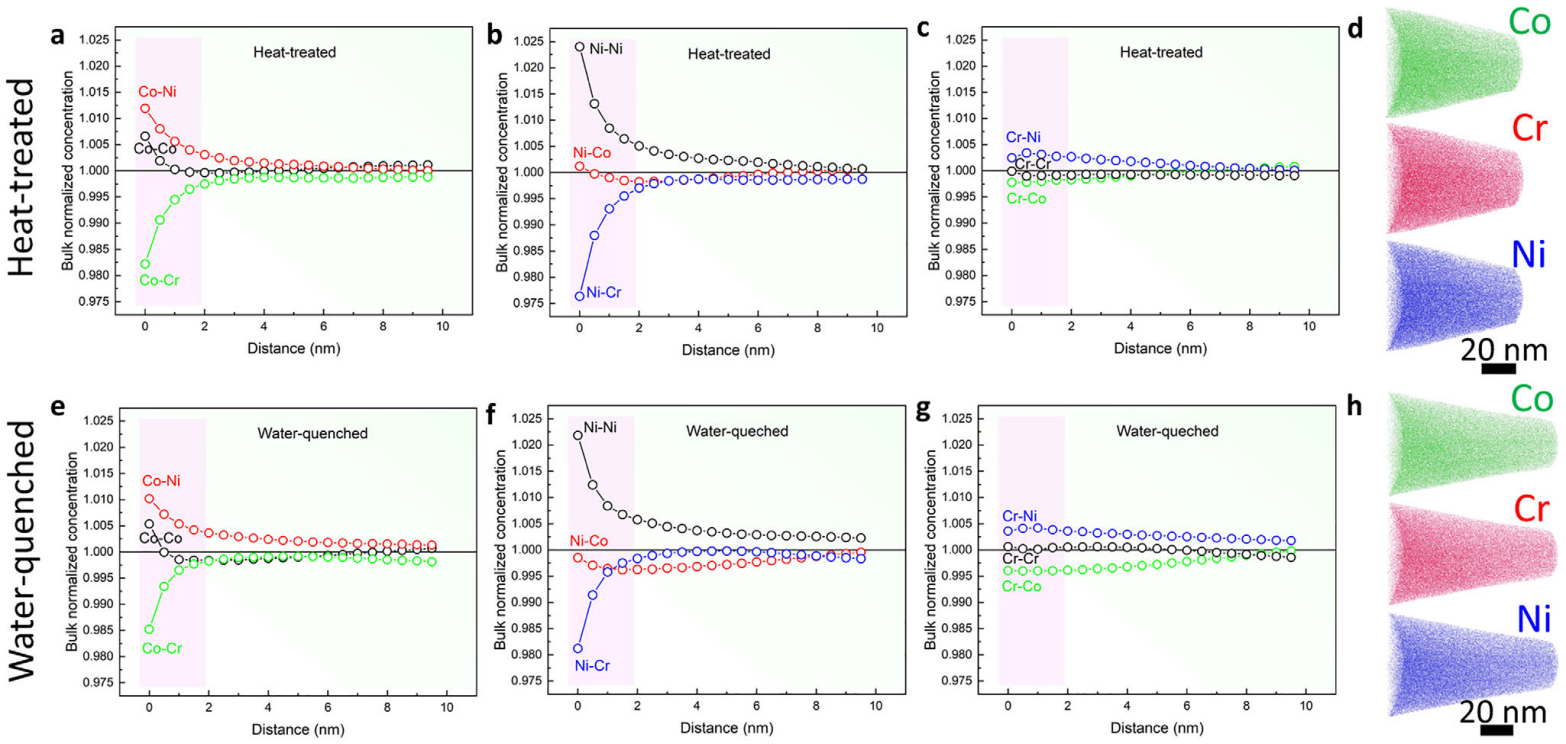
APT partial radial distribution functions (RDFs) of the heat‐treated and water‐quenched NiCoCr samples: (a, b, c) and (e, f, g) are the calculated partial RDFs from the APT data with Co, Ni, and Cr as the center atom, respectively, for heat‐treated and water‐quenched NiCoCr samples. Note that the partial RDFs were normalized by the averaged composition for the APT‐analyzed volume. (d) and (h) are APT atom maps of the constituent elements (Co, Cr, and Ni) for the two samples, respectively. This figure is reprinted from Ref. [53], licensed under CC BY 4.0.

Specifically, Hsiao and co‐authors [53] presented the reconstructed APT volumes of two samples, which indicate no signs of decomposition in Figure 8. Note that these two NiCoCr alloys are processed with water‐quenched and heat‐treated (annealed/furnace‐cooled) conditions, respectively, after a homogenization treatment. In particular, the NiCoCr alloys were prepared from high purity (> 99.9% weight percent) Cr, Co, and Ni by arc melting and subsequently drop casting under an argon atmosphere. To assure the compositional homogeneity, the melting and casting processes were repeated several times. The prepared alloys were machined into two pieces and homogenized at 1,200 °C for 48 h. Following that, one was immediately cooled by water quenching, and another was aged at 1,000 °C for 120 h and then furnace cooled to room temperature [53, 54]. A detailed investigation of the APT data, based on the partial radial distribution function (RDF) analysis, clearly indicates the presence for Ni clustering extending to few nm in radius and negative Ni‐Cr and Co‐Cr correlations. The latter suggests the Cr depletion in Ni‐ or Co‐rich clusters, while the Ni‐Co correlation in Ni‐rich clusters is somewhat negative (Figure 8b,f). Compared to Ni, the measured Cr‐Cr correlation is close to 1, with the influence of Cr‐poor and rich regions largely canceled out [53].

These studies illustrate the versatility of APT for probing a wide range of local‐ordering phenomena. Applications ranging from Fe─Al alloys [51] to oxygen‐modified TiZrHfNb alloys [52] demonstrate the ability of APT to resolve complex local chemical environments associated with both substitutional and interstitial species. These examples highlight the broad applicability of APT for characterizing local chemical order across chemically diverse MPEAs and related compositionally complex alloys.

However, APT measurements are subject to important limitations when used to quantify CSRO. The accuracy of extracted metrics depends on detector efficiency, spatial resolution, and reconstruction algorithms, which can introduce biases in nearest‐neighbor statistics. In addition, the limited sampling volume and potential reconstruction artifacts can affect the statistical reliability of measured correlations. Therefore, quantitative interpretation requires careful statistical analysis and, in many cases, validation through complementary experimental or computational approaches.

### 3.4 TEM‐Based Methods

High‐angle annular dark‐field scanning transmission electron microscopy (HAADF‐STEM) and related TEM techniques provide direct, real‐space sensitivity to local chemical variations and can be combined with statistics‐based analysis to quantify CSRO. These methods are used to visualize local chemical fluctuations and ordering motifs at the atomic scale, offering spatially resolved insight into CSRO. Xu et al. demonstrated this capability in the BCC MPEA TiVNbHf(Al), where atomic‐resolution HAADF‐STEM images were evaluated using spatial statistical tests against random and ordered reference states [55]. Their analysis showed that both alloys exhibit measurable CSRO and that Al additions amplify CSRO, illustrating how STEM image statistics can directly detect ordering motifs in refractory MPEAs.

Liu et al. applied a complementary approach to the MPEA Fe_50_Mn_30_Co_10_Cr_10_, combining nano‐beam electron diffraction with aberration‐corrected STEM imaging. They observed the diffuse diffraction intensity characteristic of non‐random atomic arrangements and mapped correlated elemental fluctuations at the nanometer scale, demonstrating pronounced CSRO in a nominally single‐phase FCC alloy and linking these motifs to mechanical response [56].

For the MPEA NiCoCr, Zhang et al. used the energy‐filtered dark‐field TEM to form images from diffuse scattering, revealing nanometer‐scale CSRO domains that grow with thermal treatment and correlate with changes in dislocation‐slip behavior and strength – ductility balance [54]. Hsiao et al. extended CSRO mapping in the two MPEA NiCoCr with water‐quenched and heat‐treated (annealed/furnace‐cooled) conditions, respectively, after the homogenization treatment, using data‐driven four‐dimensional electron diffraction (4D‐ED): principal‐component analysis and pattern matching of large diffraction data sets identified two distinct CSRO motifs, one minimizing Cr─Cr neighbors and another with Cr‐rich alternating planes, and tracked their evolution with heat treatment, directly connecting nanoscale order with deformation pathways [53].

**FIGURE 9:**
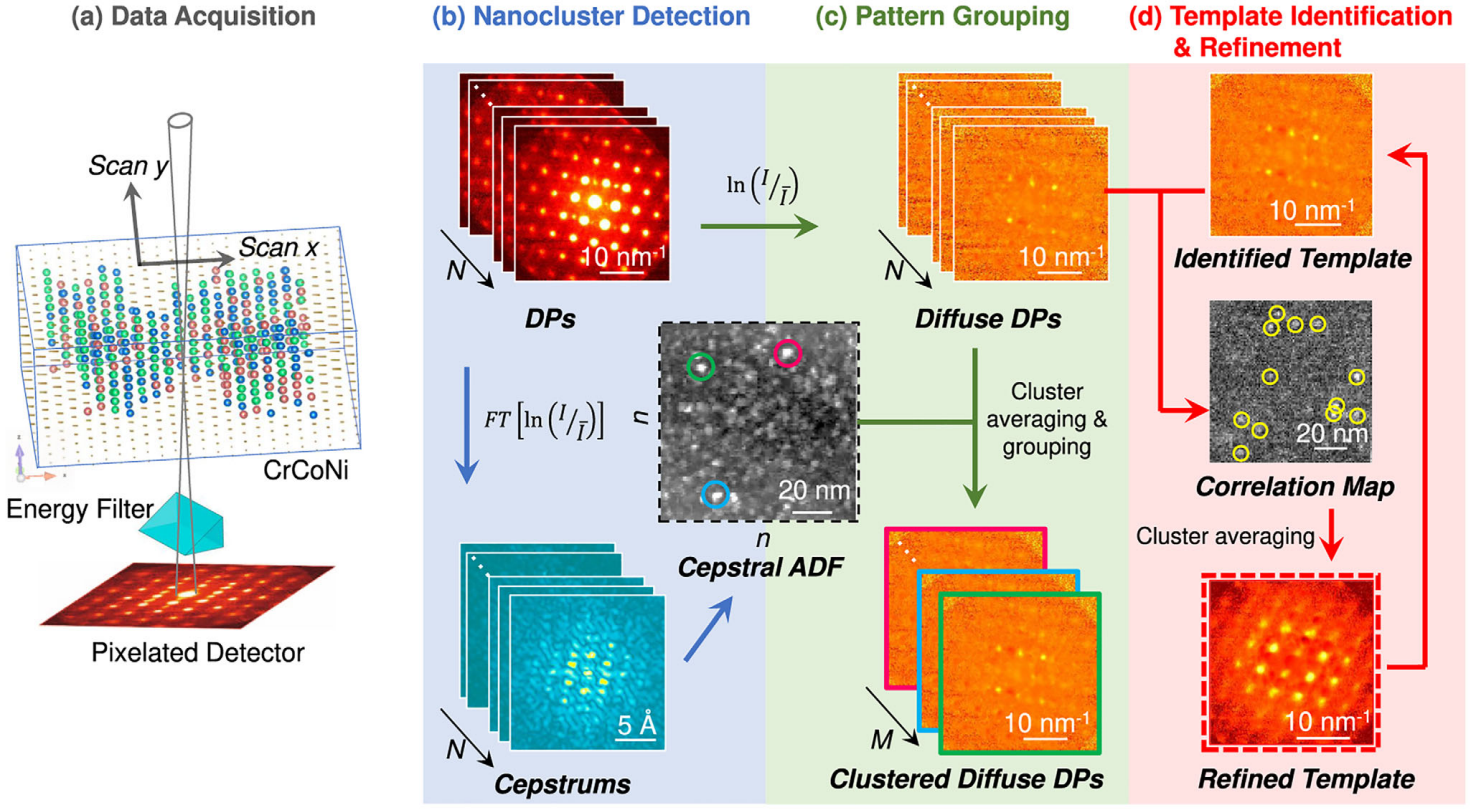
Data mining of diffraction patterns (DPs) for CSRO detection in the MPEA NiCoCr: Four steps (a–d) are included. This figure is reprinted from Ref. [53], licensed under CC BY 4.0.

Hsiao et al. [53] further found that the seemingly sameness of the two NiCoCr samples from the microstructure results presented a major puzzling question in the MEA/HEA research, e.g., the scarcity of sensitive probes within the present characterization methods for the chemical nuance in these alloys [2]. To overcome this critical issue, they established a nanodiffraction data‐mining scheme for the detection of CSRO (Figure 9). First, a nanometer‐sized convergent beam (1.1 mrad) is developed for coherent nanodiffraction. Second, an energy filter prevents the inelastic‐scattering background for diffraction‐pattern (DP) recording. Thirdly, a series of DPs is gathered in a two‐dimensional (2D) scan for a hyperspectral, four‐dimensional (4D), dataset employing a pixelated detector (Figure 9a). Data mining of the 4D dataset subsequently facilitates diffraction imaging. This powerful method, also called 4D‐STEM, has been shown for mapping strain, magnetic, and electrical fields [57, 58, 59]. The increasing application of 4D‐STEM and data‐driven diffraction analysis for mapping local structural variations [57, 58, 59, 60] also suggests potential opportunities for future investigations of local‐order evolution during thermal or mechanical loading, particularly when combined with in situ experimental platforms. In Figures 9b–d, the data‐mining process extends 4D‐STEM to diffuse‐scattering analysis. The critical point to the analysis is the cepstral STEM‐based imaging of nanoclusters (NCs) giving strong diffuse scattering [60]. Their detection subsequently facilitates an identification of diffuse patterns from these NCs. The DP grouping, template description and clarification are then utilized to assess the specific kinds of diffuse patterns and their distributions. The sensitivity of detecting CSRO can be enhanced as the extent of electron exposure is multiplied by the number of scan points, *N* ∼ 10^4^, while the spatial resolution is simultaneously strengthened from hundreds of nm in the energy‐filtered selected‐area electron diffraction (EF‐SAED) to 1 nm in scanning electron nanodiffraction (SEND).

TEM‐based methods also have some limitations for CSRO characterization. Interpretation of atomic‐scale contrast and diffuse scattering often depends on imaging conditions and data analysis methods, which can introduce ambiguity in identifying specific CSRO motifs. In addition, measurements are typically limited to small sample volumes and thin foils, which may not fully represent bulk behavior. Therefore, TEM‐based approaches are most effective when combined with complementary techniques or modeling to provide a more complete understanding of CSRO.

Recently Kim et al. [61] state that the influence of CSRO on mechanical and physical properties of MPEAs has been investigated. Nevertheless, the difficulty in unambiguously observing CSRO with high precision is truly a critical issue in developing microscopic mechanisms concerning the effects of CSRO on properties. Moreover, it is still unclear if the CSRO extends to the surfaces of MPEAs, which could alter surface‐based properties, including catalytic, oxidation, and corrosion activities. With the applications of scanning tunneling microscopy (STM), two surface CSRO domains with corresponding quasi‐long‐range orderings (QLRO) are directly observed on a MPEA (CoCrFeMnNi) surface [61]. Density functional theory (DFT) calculations discover the CSRO in QLRO supercells. These results provide evidence of the presence of the surface CSRO and demonstrate a technique to directly observe the surface CSRO of MPEAs. Their study paves the pathway toward fundamentally understanding how CSRO impacts surface‐based properties, facilitating the design of MPEAs with desirable properties.

### 3.5 Resistivity Measurements

**FIGURE 10:**
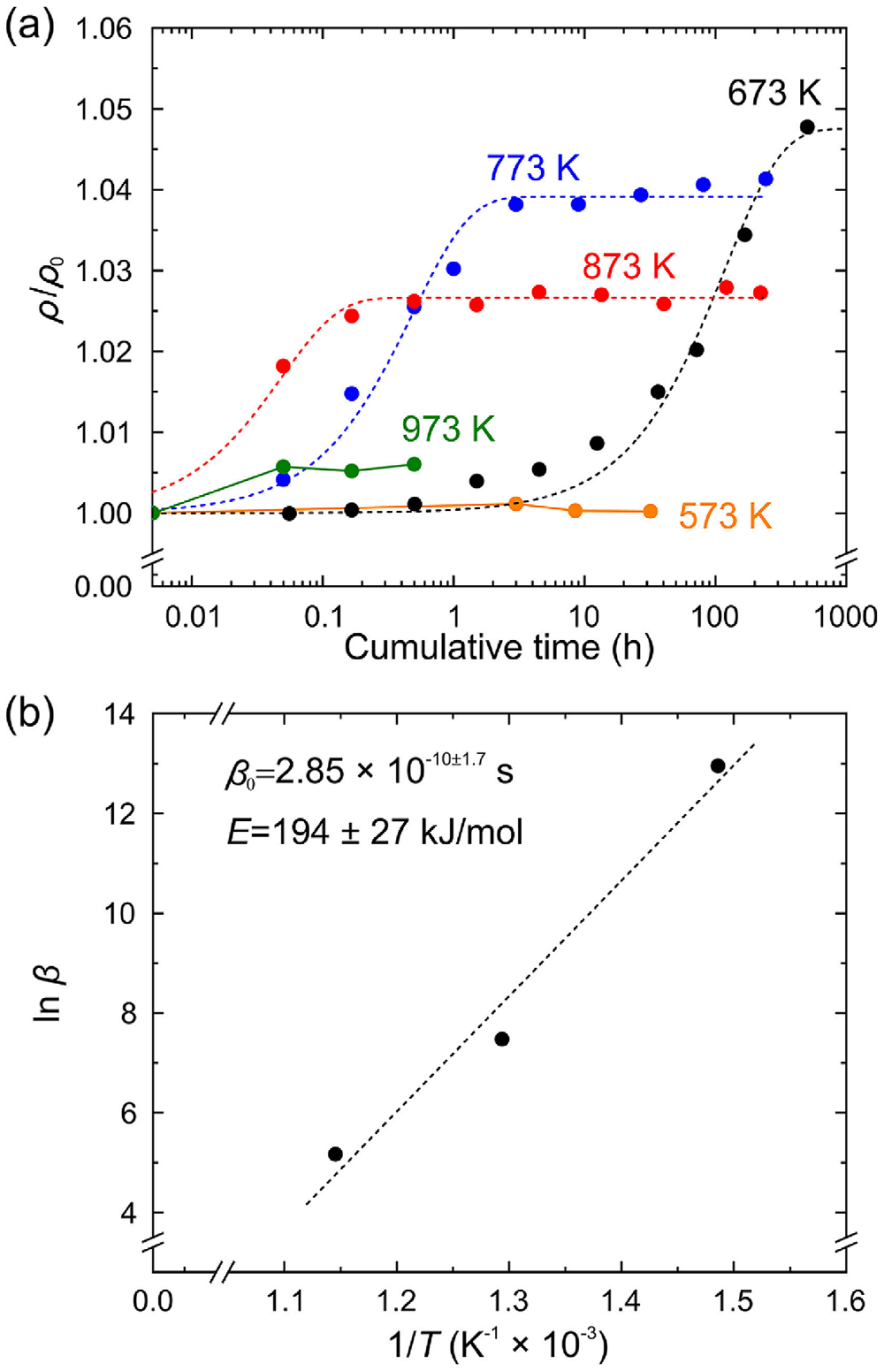
(a) Variations in electrical resistivity of the equiatomic NiCoCr polycrystals quenched from 1,473 K during isothermal annealing at various temperatures in a range from 573 to 973 K. (b) Arrhenius plot of relaxation time, *β*, for the resistivity change by isothermal annealing. This figure is reprinted from Ref. [64], with permission from Elsevier.

The electrical‐resistivity method is an extremely sensitive, indirect method employed to monitor changes associated with the evolution of CSRO. Resistivity measurements are considered because of their precision and simplicity, in particular when monitoring ordering characteristics during heat treatments. Resistivity varies since the local arrangement of atoms changes the scattering condition for conduction electrons [62, 63, 64]. Li, et al. [64] reported that the CSRO evolution in the MPEA NiCoCr was investigated by electrical‐resistivity measurements, as presented in Figure 10. Figure 10a presents the normalized room‐temperature electrical resistivities (*ρ*/*ρ*_0_) as a function of annealing time, where *ρ*_0_  =  0.028161 ± 0.00006 Ω is the resistivity of samples quenched from 1,473 K. The NiCoCr was first quenched from 1,473 K and subsequently isothermally annealed for various times at temperatures covering from 573 to 973 K. The CSRO below 973 K can be detected through variations in resistivity, which increase with time to a saturation value during isothermal annealing in the temperature range of 673 – 973 K. While the time required to reach saturation is shorter at greater temperatures, the saturation resistivity is larger at lower temperatures, suggesting a greater extent of CSRO at lower temperatures even though it requires longer time to approach saturation owing to slower kinetics.

Resistivity measurements of Figure 10a appear to provide an appropriate method to monitor CSRO in the NiCoCr in a quantitative manner. The kinetic Monte‐Carlo calculations employing potentials developed through the neural networks by Du et al. [27] have indicated that the variations in the Warren‐Cowley parameters of the NiCoCr following aging are similar to those in resistivity shown in Figure 10a with the saturated Warren‐Cowley parameter close to a value of – 0.217 at 700 K [64]. Moreover, in Figure 8a, the time‐dependent variation of resistivity at 673, 773, and 873 K could be quantitatively described by the following two equations [64],

$$\rho \left(t\right)/\;\rho_{0}=1+\delta \left\{1-\exp\left(-t/\beta \right)\right\}$$

$$\beta =\beta_{0}\;\exp\left(E/RT\right)$$

where *t* is the annealing time, and *δ* and *β* are the constants depending on the annealing temperature. *β*_o_  is a pre‐exponential factor, *R* the gas constant, *T* the absolute temperature and *E* the activation energy. The results of *β* vs. 1/T (temperature) are plotted in Figure 9b with *β_o_*  = 2.85 × 10^−10 ± 1.7^ s, and activation energy (*E* = 194 ± 27 kJ/mol).

The resistivity measurements provide indirect evidence of CSRO and do not uniquely determine specific atomic configurations. Changes in resistivity can also be influenced by other factors, such as defects, impurities, and phase transformations, making it difficult to isolate the contribution of CSRO alone. Therefore, resistivity‐based assessments of CSRO should be interpreted in conjunction with complementary experimental techniques or modeling to ensure reliable conclusions.

This figure is reprinted from Ref. [47], with permission from the American Physical Society.

Table 2 summarizes the experimental tools available for the study and characterization of CSRO, together with their principal strengths and limitations. Similar to computational approaches, these experimental techniques provide complementary rather than redundant information regarding local chemical order. Scattering‐ and spectroscopy‐based methods, such as PDF, EXAFS, and XANES, probe local atomic correlations and bonding environments, while atom probe tomography and advanced electron microscopy techniques provide direct insights into local chemical distributions and atomic‐scale structural heterogeneities. Other approaches, including diffraction‐based measurements and transport‐property characterization, often serve as indirect probes of CSRO evolution and its influence on material behavior. Because each technique is sensitive to different length scales, time scales, and manifestations of local order, no single experimental method can fully characterize CSRO in complex alloys. Consequently, reliable quantification and interpretation of CSRO increasingly require integrated characterization and modeling strategies that combine multiple experimental observables and, when possible, leverage computational predictions for validation and interpretation.

**TABLE 2:** Experimental techniques for characterizing CSRO in MPEAs.

Method	Resolution	Key Advancements	Limitations	References
Total scattering and PDF	0.1 – 10 Å (real‐space)	Measures real‐space pair correlations; sensitive to CSRO and local distortions; complements RMC refinement	Requires neutron/synchrotron sources; PDF solutions can be non‐unique	[40, 41, 42, 43]
In situ high‐energy synchrotron X‐ray diffraction	∼ 1 – 5 µm (gauge volume)	Tracks ordering/phase evolution during annealing/solidification; provides kinetic constraints for CSRO formation	Limited sensitivity to very short‐range correlations; diffuse intensity often weak	[44, 45, 46, 47, 48]
Atom probe tomography	< 0.5 nm (3D)	3D atomic‐scale composition; enables quantitative CSRO through nearest‐neighbor statistics; ML reconstruction enhances fidelity	Small sample volume; reconstruction artifacts; requires careful statistical treatment	[49, 50, 51, 52, 53, 54]
TEM‐based methods	< 0.1 nm (atomic columns)	Direct imaging of local chemical variations; diffraction‐based mapping of CSRO motifs; correlates CSRO with strain and defects; STM enables detection of surface CSRO	Thin‐foil requirement; beam sensitivity; limited to local regions; STM requires high‐quality surface preparation and surface integrity	[53, 54, 55, 56, 57, 58, 59, 60, 61]
Resistivity Measurements	N/A	Correlates resistivity with CSRO	Indirect measure of CSRO; interpretation may be complicated by competing scattering mechanisms	[62, 63, 64]

An important consideration across all experimental approaches is that the detectability of CSRO depends not only on the magnitude of the underlying chemical correlations, but also on instrumental and statistical limitations. Although quantitative detection thresholds are rarely universal, the sensitivity of each technique depends strongly on instrument performance, data quality, and the magnitude of the underlying chemical correlations. For example, the sensitivity of atom probe tomography is influenced by detector efficiency, spatial resolution, reconstruction accuracy, and sampling statistics, while the detectability of CSRO through total‐scattering and PDF‐based techniques depends on reciprocal‐space coverage, counting statistics, and the strength of the ordering‐induced structural signatures. Similarly, resistivity measurements provide indirect evidence of CSRO and require careful separation of ordering effects from other contributions to electron scattering. Consequently, the practical detection limits of CSRO are highly system‐ and method‐dependent, reinforcing the need for complementary experimental observables and computational predictions when assessing local chemical order.

## 4 Modeling–Experiment Cross‐Validation Pathways for CSRO in MPEAs

Cross‐validating CSRO formation in MPEAs is inherently different from verifying a phase diagram or a microstructure map. CSRO is not a single, uniquely recoverable structure. It is a statistical bias in local environments that leaves signatures across multiple observables. For that reason, strong validation rarely comes from one probe matched to one model output. Instead, confidence builds when independent measurements constrain the same underlying ordering trends in compatible ways. This reflects the fundamentally non‐unique nature of CSRO characterization, where multiple atomic configurations may produce similar experimental signatures.

A practical starting point is to connect atomistic CSRO descriptors to scattering observables. Modeling outputs, such as partial pair‐correlation functions and Warren–Cowley parameters, can be compared indirectly against total‐scattering and pair‐distribution‐function (PDF) features, especially the first two to three coordination shells where CSRO effects are concentrated. PDF‐based analyses demonstrate sensitivity to subtle deviations from an average random lattice and show that cluster‐based structural representations reproduce short‐range PDF behavior better than average crystal models [40, 43]. Although such comparisons are rarely unique, they impose strong constraints on physically meaningful CSRO states.

Real‐space probes provide a complementary validation channel. Predictions of preferred atomic pairings, clustering tendencies, or layered motifs can be assessed through APT using nearest‐neighbor statistics and partial radial distribution function analyses, provided that detectability limits are respected [49]. Studies on NiCoCr reveal non‐random correlations and nanoscale chemical modulations, while also illustrating the need for careful statistical interpretation [50, 53]. Advanced TEM‐based approaches, including diffuse‐scattering imaging and data‐mined nanodiffraction or four‐dimensional STEM, further enable mapping of CSRO motifs and their evolution with heat treatment [53, 54, 57, 58, 59, 60]. Surface CSRO has been detected through STM [61].

Time‐dependent measurements add a third axis for validation. Electrical resistivity measurements sensitively track changes in local chemical environments during isothermal annealing [61, 62, 63]. In NiCoCr, the evolution of resistivity has been quantitatively linked, at the level of activation energies and time constants, to KMC simulations using neural‐network‐derived potentials, showing consistent activation energies and saturation behavior between the experiment and modeled CSRO evolution [27].

**FIGURE 11:**
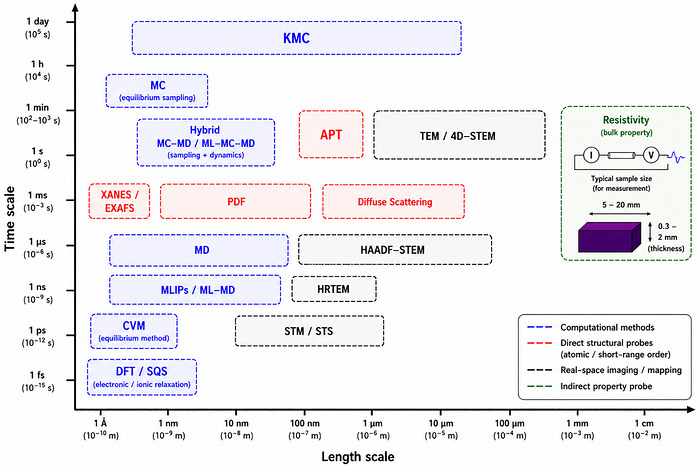
Characteristic length scales and time scales accessible to computational and experimental approaches used for studying CSRO in multi‐principal element alloys. Computational methods (blue), direct characterization techniques (red and black), and indirect property‐based probes (green) are positioned according to their typical spatial and temporal domains. Overlapping regions indicate opportunities for cross‐validation among complementary approaches, while sparsely populated regions highlight current challenges in bridging atomistic mechanisms with experimentally relevant length and time scales. Dashed boundaries indicate that the spatial and temporal ranges shown are approximate and method‐dependent; in practice, many approaches can extend beyond the indicated domains depending on the specific implementation, system size, instrumentation, and computational resources.

The opportunities for cross‐validation among computational and experimental approaches are further illustrated in Figure 11, which compares the characteristic spatial and temporal domains accessible to the principal methods used to study CSRO. The figure highlights the complementary nature of these approaches, as different methods probe distinct observables and operate across different length and time scales. While first‐principles calculations, statistical thermodynamics, and atomistic simulations provide insight into the formation and evolution of local chemical order, experimental techniques probe its structural, chemical, and property‐related manifestations through different observables. The overlap among these approaches enables cross‐validation of CSRO descriptors and interpretations, whereas sparsely covered regions highlight remaining challenges in connecting atomistic mechanisms with experimentally relevant length and time scales.

Finally, mechanistic validation requires consistency between CSRO‐modified defect energetics predicted by atomistic simulations and experimentally observed deformation behavior. Hybrid MC–MD studies demonstrate that CSRO roughens the dislocation energy landscape, alters stacking‐fault energies, and promotes intermittent pinning [22, 33, 34]. Agreement with microscopy‐based observations of slip behavior and local ordering motifs under comparable processing conditions strengthens confidence in these CSRO‐informed mechanisms [53, 54].

The examples discussed above illustrate that robust validation of CSRO rarely relies on a single computational prediction or experimental measurement. Instead, confidence emerges when multiple independent observables consistently support the same underlying ordering trends. Table 3 summarizes representative cross‐validation pathways for connecting common CSRO predictions with complementary computational and experimental approaches, together with the criteria used to assess consistency between modeled and measured behavior.

**TABLE 3:** Cross‐validation of CSRO predictions using combined computational and experimental approaches in MPEAs.

CSRO prediction	Computational approach (references)	Experimental approach (references)	Cross‐validation criteria
Preferred atomic pair correlations (Warren–Cowley–type pair preferences)	SQS + DFT; DFT‐based cluster energetics [9, 10, 11, 12, 13, 14, 15, 16, 17, 18]; MC sampling of pair correlations [27, 28, 29, 30, 31, 32, 33]	Total scattering and PDF analysis [40, 41, 42, 43]	Predicted pair‐preference trends are consistent with short‐range PDF peak evolution (first two to three coordination shells) and associated diffuse‐scattering signatures
Temperature‐dependent CSRO formation and stability trends	CVM; cluster‐based thermodynamics and CALPHAD‐integrated CSRO models [21, 22, 23, 24, 25, 26]	In‐situ high‐energy synchrotron XRD, and EXAFS [44, 45, 46, 47, 48]	Predicted ordering tendencies and stability windows remain compatible with experimentally observed time–temperature evolution of phase and local‐bond signatures
Equilibrium CSRO motifs and local chemical environments	Monte Carlo sampling of motifs and correlations [27, 28, 29, 30, 31, 32, 33]	PDF analysis; APT nearest‐neighbor and partial RDF statistics [40, 41, 42, 43, 49, 50, 51, 52, 53, 54]	Dominant motif‐level correlation trends are consistent across scattering‐based and real‐space statistics, acknowledging non‐uniqueness and sampling limitations
CSRO evolution kinetics (time constants and activation energies)	Kinetic Monte Carlo using neural‐network‐derived potentials [27]	Electrical‐resistivity measurements during isothermal annealing [62, 63, 64]	Comparable activation energies, saturation behavior, and temperature dependence of ordering kinetics
CSRO‐modified defect energetics (stacking‐fault energy, dislocation pinning)	Molecular dynamics and hybrid MC–MD simulations [22, 31, 33, 34]	TEM‐based characterization of slip behavior and local ordering motifs [53, 54]	Predicted changes in defect energetics and deformation mechanisms remain compatible with observed dislocation behavior under comparable processing conditions
Large‐scale CSRO trends across composition space	Machine‐learning approaches (GNNs, MLIPs, DeepMD, and PINNs) [31, 32, 35, 36, 37, 38, 39]	APT, TEM, 4D‐STEM diffuse‐scattering and motif mapping, and STM [49, 50, 51, 52, 53, 54, 57, 58, 59, 60, 61]	Statistically consistent motif distributions and compositional trends across length scales, with discrepancies informing model refinement and dataset expansion

While the specific validation pathway depends on the material system and the nature of the predicted ordering behavior, a common workflow begins with computational prediction of CSRO descriptors, such as pair correlations, motif distributions, or ordering kinetics, followed by selection of experimental observables that are sensitive to the corresponding signatures of local chemical order. Quantitative comparison between predicted descriptors and experimentally derived observables then enables cross‐validation of ordering trends, stability, kinetics, and defect‐related mechanisms. Discrepancies between predictions and measurements can further guide refinement of computational models, experimental analyses, or both, establishing an iterative framework for improving the reliability of CSRO characterization and interpretation.

## 5 Challenges and Future Directions

A central challenge moving forward is the development, generalization, and systematic validation of CSRO models that can reliably link processing conditions to CSRO and, ultimately, to mechanical and functional properties in MPEAs. Although recent studies demonstrate that meaningful cross‐validation between modeling and experiments is feasible in selected systems, establishing such validation in a robust, transferable, and predictive manner remains an open problem. CSRO arises from a delicate balance of thermodynamic driving forces, kinetic constraints, and local compositional fluctuations, and capturing this balance across alloy families and processing pathways continues to pose fundamental challenges. As a result, quantitative Process–Structure–Property (PSP) relationships that explicitly incorporate CSRO are still in an early stage of development. A key underlying challenge is that CSRO cannot be uniquely determined from any single modeling or experimental approach, requiring integrated, multi‐modal frameworks to achieve reliable interpretation and prediction.

From a modeling perspective, a major challenge lies in bridging scales in a way that preserves physically meaningful CSRO descriptors. Atomistic approaches, including DFT, Monte Carlo, molecular dynamics, and emerging ML‐based frameworks, have provided important insights into CSRO energetics, motifs, and kinetics. These approaches have also provided valuable insight into the interactions among local chemical order, defect activity, microstructural evolution, and materials behavior. Recent studies have further emphasized the importance of understanding these interactions in governing deformation behavior, phase transformations, and microstructural evolution in compositionally complex alloys [65, 66]. However, their predictions remain sensitive to assumptions regarding interaction parameters, sampling strategies, and training data, particularly in multicomponent composition spaces. ML‐accelerated atomistic simulations, including physics‐informed neural networks, equivariant graph‐based models, and deep‐learning interatomic potentials, offer a promising route to explore broader processing histories and chemical spaces. Nevertheless, ensuring transferability, interpretability, and uncertainty quantification in these models remains a critical open challenge, especially when extrapolating beyond well‐studied systems, such as NiCoCr.

Another major open challenge is the incorporation of CSRO into mesoscale and continuum frameworks. While phase‐field modeling has successfully captured precipitate morphologies, elastic interactions, and multiphase evolution in MPEAs, the explicit inclusion of CSRO‐aware thermodynamics is still limited. Developing phase‐field formulations that are informed by atomistically resolved CSRO energetics, while remaining computationally tractable at mesoscopic length and time scales, is an unresolved problem. Progress in this area will require careful integration of cluster‐based thermodynamic descriptions, validated interaction parameters, and appropriate treatments of kinetics and elasticity. Without such developments, linking processing parameters to CSRO‐driven microstructural evolution will remain largely qualitative.

On the experimental side, significant challenges persist in directly and unambiguously characterizing CSRO and its evolution under realistic processing and service conditions. Individual techniques, such as total scattering, high‐energy diffraction, APT, and advanced TEM‐based methods, each provides partial and complementary views of CSRO, but none alone can uniquely resolve CSRO. Developing coordinated in situ and operando experimental platforms that synchronize these probes under controlled thermal, mechanical, or irradiation stimuli remains an open frontier. Such efforts are essential not only for observing dynamic CSRO pathways, but also for providing the statistically robust datasets required to constrain and test predictive models. Particularly important is the development of experimental approaches capable of directly probing dynamically evolving local order under external stimuli, thereby enabling rigorous validation of emerging computational predictions.

Equally important are challenges associated with integration and standardization. Differences in the spatial resolution, sampling volume, statistical metrics, and data representation across experimental techniques complicate direct comparisons with modeling outputs. Establishing consistent protocols for quantifying CSRO, harmonizing data formats, and defining reproducible workflows for model calibration and cross‐validation are critical unmet needs. Addressing these issues is a prerequisite for moving from case‐by‐case validation studies toward broadly applicable, CSRO‐informed design strategies.

Finally, improving the integration of modeling and experiments through rigorous cross‐validation remains a major need, particularly in the presence of inherent uncertainty and non‐uniqueness in CSRO characterization. Establishing consistent protocols for quantifying CSRO across techniques, harmonizing data formats, and defining robust workflows for iterative cross‐validation, calibration, and refinement of models against experimental observations will be crucial for enabling predictive, CSRO‐informed alloy design. Ultimately, achieving reliable PSPP relationships requires a unified framework where modeling, characterization, and data analytics converge to capture how processing governs CSRO, how CSRO shapes nano‐/micro‐structures, and how these structures dictate properties and macroscopic performance.

## Conflicts of Interest

The authors declare no conflicts of interest.

## Data Availability

Data sharing not applicable to this article as no datasets were generated or analysed during the current study.
